# Niacin supplementation attenuates the regression of three‐dimensional capillary architecture in unloaded female rat skeletal muscle

**DOI:** 10.14814/phy2.16019

**Published:** 2024-04-16

**Authors:** Hao Lin, Jihao Xing, Han Pan, Takumi Hirabayashi, Noriaki Maeshige, Ryosuke Nakanishi, Hiroyo Kondo, Hidemi Fujino

**Affiliations:** ^1^ Department of Rehabilitation Science Kobe University Graduate School of Health Sciences Kobe Japan

**Keywords:** capillary regression, mitochondrial metabolism, muscle atrophy, niacin

## Abstract

Inactivity can lead to muscle atrophy and capillary regression in skeletal muscle. Niacin (NA), known for inducing hypermetabolism, may help prevent this capillary regression. In this study involving adult female Sprague–Dawley rats, the animals were randomly assigned to one of four groups: control (CON), hindlimb unloading (HU), NA, and HU with NA supplementation (HU + NA). For a period of 2 weeks, the rats in the HU and HU + NA groups underwent HU, while those in the NA and HU + NA groups received NA (750 mg/kg) twice daily through oral administration. The results demonstrated that HU lowered capillary number, luminal diameter, and capillary volume, as well as decreased succinate dehydrogenase activity, slow fiber composition, and PGC‐1α expression within the soleus muscle. However, NA supplementation prevented these alterations in capillary structure due to unloading by stimulating PGC‐1α factors and inhibiting mitochondrial dysfunction. Therefore, NA supplementation could serve as a potential therapeutic approach for preserving the capillary network and mitochondrial metabolism of muscle fibers during periods of inactivity.

## 1 INTRODUCTION

Capillaries play a vital role in delivering oxygen and nutrients to skeletal muscles and are susceptible to structural alterations in response to variety of physiological circumstances (Olfert & Birot, 2011). Physical inactivity is known to cause capillary degeneration (Fujino et al., 2005). Specifically, Hindlimb‐unloading (HU) are known as rodent inactivity model, can lead to capillary regression due to reduced oxygen demand (Roudier et al., 2010). Furthermore, capillary regression impairs exercise capacity by decreasing maximal oxygen uptake (VO_2_) (Tadaishi et al., 2011). Therefore, it is crucial to prevent capillary regression during HU conditions. While exercise is an effective method for preventing capillary regression in impaired muscles (Fujino et al., 2014), nutritional therapy should also be considered for faster rehabilitation in bedridden patients.

Niacin (NA), an essential human nutrient and a form of vitamin B3, is involved in the nicotinamide adenine dinucleotide (NAD) cycle. Supplementation with NA (750 mg/kg/day for 4 weeks) induced the transition from glycolytic (fast muscles) to oxidative muscle (slow muscles) fibers by upregulating mitochondrial biogenesis in obese Zucker rats (Ringsei et al., 2013). The oxidative muscles have a greater number of capillaries and tortuosity than glycolytic muscles, suggesting improved capillary health (Fujino et al., 2012). Additionally, high levels of NA supplementation upregulate transcription factors like PGC‐1α in growing pigs, as reported by Khan et al. (2013). In skeletal muscle cells, PGC‐1α strongly induces mitochondrial biogenesis, and enhances energy metabolism within muscle fiber mitochondria. (Arany et al., 2008; Nakazato & Song, 2008) This increased mitochondrial metabolic activity facilitates elevated oxygen supply to the soleus muscles, leading to angiogenesis in the skeletal muscle. (Charifi et al., 2004) Thus, we hypothesized that administering NA to hindlimb‐unloaded rats would attenuate mitochondrial dysfunction within the unloaded muscle, thereby protecting against capillary regression and promoting the remodeling of capillary networks associated with muscle atrophy.

Capillary network remodeling in skeletal muscle involves changes in three‐dimensional (3D) structures. (Fujino et al.,^2005^) In particular, a decrease in capillary diameter is associated with an increase in the prevalence of dysfunctional capillaries measuring less than 2.5 μm in diameter, making it important to assess these in 3D rather than 2D. (Henquell et al., 1977). Hence, it is necessary to analyze using 3D methods for comprehensive analysis of capillary remodeling. In this study, we investigated the effect of NA supplementation following unloading for capillary regression using 3D capillary architecture analysis in hindlimb‐unloaded rat skeletal muscles.

## 2 MATERIALS AND METHODS

### 2.1 Experimental groups

In this study, 24 female Sprague–Dawley rats were used. The rats, with an average weight of 308.3 ± 4.7 g, were purchased from Japan SLC (Hamamatsu, Japan). After a one‐week acclimatization period, the rats were randomly divided into four groups: control (CON, *n* = 6), NA supplementation (NA, *n* = 6), 14‐day HU (HU, *n* = 6), and NA supplementation during HU (HU + NA, *n* = 6). Rats in the NA and HU + NA groups received oral administration of NA (146–01235, Wako Pure Chemical Industries, Osaka, Japan) (750 mg/kg/day) dissolved in a 5% arabic gum solution (016–00025, Wako Pure Chemical Industries) via a catheter twice daily. Rats in the other groups received the same volume of 5% gum arabic solution, which served as an emulsifier. After 1 week of administration for acclimatization, the rats in the HU and HU + NA groups underwent HU for 2 weeks. All rats received either the solution or NA during the HU period. The rats were housed in an isolated, environmentally controlled room at a temperature of 22 ± 2°C, with a 12‐h light–dark cycle. They had *ad libitum* access to food (Diet CE‐2, CLEA Japan, Tokyo, Japan, Table 1) and drinking water.

**TABLE 1:** Composition of the experimental diets.

Nutritional component/100 g CE‐2	
Moisture (%)	8.9
Crude protein (%)	24.9
Crude fat (%)	4.6
Crude fiber (%)	4.1
Crude ash (%)	6.6
NFE (%)	51.0
Energy (kcal)	345

### 2.2 HU

HU was performed according to the procedure previously described by (Morey et al., 1979). In brief, each rat was suspended by its tail using adhesive tape and a kite string, preventing their hindlimbs from touching the floor and sides of the cage. The forelimbs were allowed to maintain contact with the cage floor.

### 2.3 Sample preparation

Approximately 12 h after the final oral administration, all rats were deeply anesthetized with pentobarbital (50 mg/kg/i.p.). Soleus muscles were then removed and weighed. The muscles were immediately frozen in an acetone‐dry ice bath and stored at −80°C until analysis.

### 2.4 Histological analysis

Transverse soleus muscle sections, 10 μm thick, were cut on a cryostat (CM‐1510S, Leica Microsystems, Mannheim, Germany) at −25°C and mounted on glass slides. The sections were stained for myofibrillar adenosine triphosphatase (ATPase) after preincubation at pH 4.2 and alkaline phosphatase to identify muscle fiber types and capillaries, respectively. Stained sections were used to measure fiber cross‐sectional area (FCSA), slow fiber composition, and count the number of capillaries and muscle fibers to calculate the capillary‐to‐fiber (C/F) ratio using the Image J software program (NIH, Bethesda, MD). Staining for succinate dehydrogenase (SDH), which is used as an oxidative marker, was conducted according to previously described methods (Kanazashi et al., 2019). Briefly, sections were stained in a solution containing 0.1 M phosphate buffer (pH 7.6), 0.9 mM NaN3, 0.9 mM 1‐methoxyphenazine methylsulfate (M2077, LKT Laboratories, St. Paul, Minnesota), 1.5 mM nitroblue tetrazolium (298–83‐9, Nacalia Tesque, Kyoto, Japan), 5.6 mM EDTA‐disodium salt (6381‐92‐6, MP Biomedicals, Inc.), and 48 mM succinate disodium salt (32405–75, Nacalia Tesque) for 45 min at 37°C. The sections were then dehydrated in graded ethanol solutions and dipped in xylene. Densitometric analysis of SDH activity was performed using ImageJ software and reported as a percentage of the control group.

### 2.5 3D analysis of capillary architecture

Following established techniques, capillary volume and luminal diameter in the right soleus muscle were measured using 3D imaging methods (Fujino et al., 2005, 2012). We applied this method to visualize skeletal muscle capillaries in the following manner. In brief, 200 μm thick longitudinal sections were scanned to a depth of 50 μm using a BZ‐X810 fluorescence digital microscope (Keyence, Osaka, Japan). Sequential Z stacks were recorded with an optical sectioning pitch of 1.2 μm. Z stacks were 3‐dimesionally reconstructed using Keyence ImageViewer (software version, BZ‐X800 Viewer, Keyence, Osaka, Japan) equipped with a Plan Apochromat 10x objective (NA0.45, BZ‐PA10, Keyence). The 41‐slice stack that resulted was digitally processed in order to visualize the 3D capillary network architecture. Finally, capillary volume was quantified from a 3D image (250 × 250 × 50 μm, length × width × depth), while capillary luminal diameter (in micrometers, μm) was assessed using the Image J software program (NIH, Bethesda, MD, USA).

### 2.6 SDS polyacrylamide gel electrophoresis and western blot analysis

The muscles were homogenized on ice in a homogenization buffer (20 mM HEPES) (pH 7.4), 75 mM NaCl, 2.5 mM MgCl2, 0.1 mM EDTA, 0.05% (vol/vol) Triton X‐100, 20 mM beta‐glycerophosphate, 1 mM Na3VO4, 10 mM NaF, 0.5 mM DTT, and 1% (vol/vol) protease inhibitor cocktail for mammalian tissue (Sigma‐Aldrich, St. Louis, MO, USA). The homogenates were centrifuged at 1700*g* for 10 min at 4°C, and supernatants were collected. The total protein concentration of the supernatants was determined as previously described. The supernatants were solubilized in sample loading buffer, which contained 50 mmol/L Tris–HCl pH 6.8, 2% sodium dodecyl sulfate, 10% glycerol, 5% 2‐mercaptoethanol, and 0.005% bromophenol blue. The samples were boiled for 10 min at 80°C.

Proteins (30 μg/lane) were separated by SDS polyacrylamide gel electrophoresis (SDS‐PAGE) and transferred onto polyvinylidene fluoride (PVDF) membranes. The membranes were blocked for 1 h in 5% skimmed milk in phosphate‐buffered saline with Tween‐20 (PBST). Following blocking, the membranes were incubated with primary antibodies, anti‐PGC‐1α (1:200 in PBST, sc‐13,067; Santa Cruz Biotechnology, Santa Cruz, CA), and anti‐GAPDH (1:1000 in PBST, sc‐32,233; Santa Cruz Biochemistry). The primary antibodies were detected by anti‐rabbit IgG (HAF008, R&D Systems, Northeast, Minneapolis) or anti‐mouse IgG (HAF007, R&D Systems.) conjugated to horseradish peroxidase (GE Healthcare, Waukesha, WI), visualized using a chemiluminescent reagent (Ez West Lumi, ATTO, Tokyo, Japan), and determined using an image reader (LAS‐1000, Fujifilm, Tokyo, Japan).

### 2.7 Citrate synthase (CS) activity analysis

CS activity was determined using previously published methods (Srere & Bhaduri, 1964). In summary, supernatants were solubilized in a reaction buffer comprising 0.1 mM DTNB (D8130‐500MG, Sigma‐Aldrich, St. Louis, MO) and 0.3 mM acetyl‐CoA (014–10,813, Wako Pure Chemical Industries). The reaction was initiated with 0.5 mM oxaloacetic acid (014–10,813, Wako Pure Chemical Industries), and absorbance was monitored at 412 nm for 5 min.

### 2.8 Statistical analysis

All data are presented as means ± SEM. Differences were assessed by one‐way ANOVA followed by Tukey–Kramer's post hoc test to determine specific group differences. A significance level of *p* < 0.05 was set for all tests.

## 3 RESULTS

### 3.1 Body, muscle mass and fiber cross‐sectional areas

The muscle mass and FCSA in the HU and HU + NA groups were significantly lower than those in the control group (Table 2). However, there was no difference among the four groups regarding body mass.

**TABLE 2:** Bodyweight, soleus weight and soleus muscle FCSA.

	Body weight (*g*)	Soleus muscle weight (mg)	Soleus muscle FCSA (μm^2^)
CON	299.7 ± 25.3	145 ± 19.1	2375.9 ± 496.7
NA	289.5 ± 25.9	132 ± 17.6	2629.8 ± 273.6
HU	271.5 ± 22.2	90 ± 10.7*†	1030.9 ± 32.6*†
HU + NA	269.2 ± 22.2	104 ± 14.7*†	1592.9 ± 141.7*†‡

*Note*: Values are means ± SD (*n* = 6). The symbols *, †and ‡ indicate significant differences from the CON, NA and HU groups, respectively, at *p* < 0.05. Unloading (HU and HU + NA) resulted in a decrease in the soleus muscle weight and soleus muscle FCSA. However, NA treatment attenuated the HU‐induced reduction of FCSA.

Abbreviations: CON, control; HU, hindlimb unloaded; NA, Niacin; HU + NA, hindlimb unloaded plus Niacin.

### 3.2 Muscle fiber morphometry and distribution of fiber sizes

**FIGURE 1:**
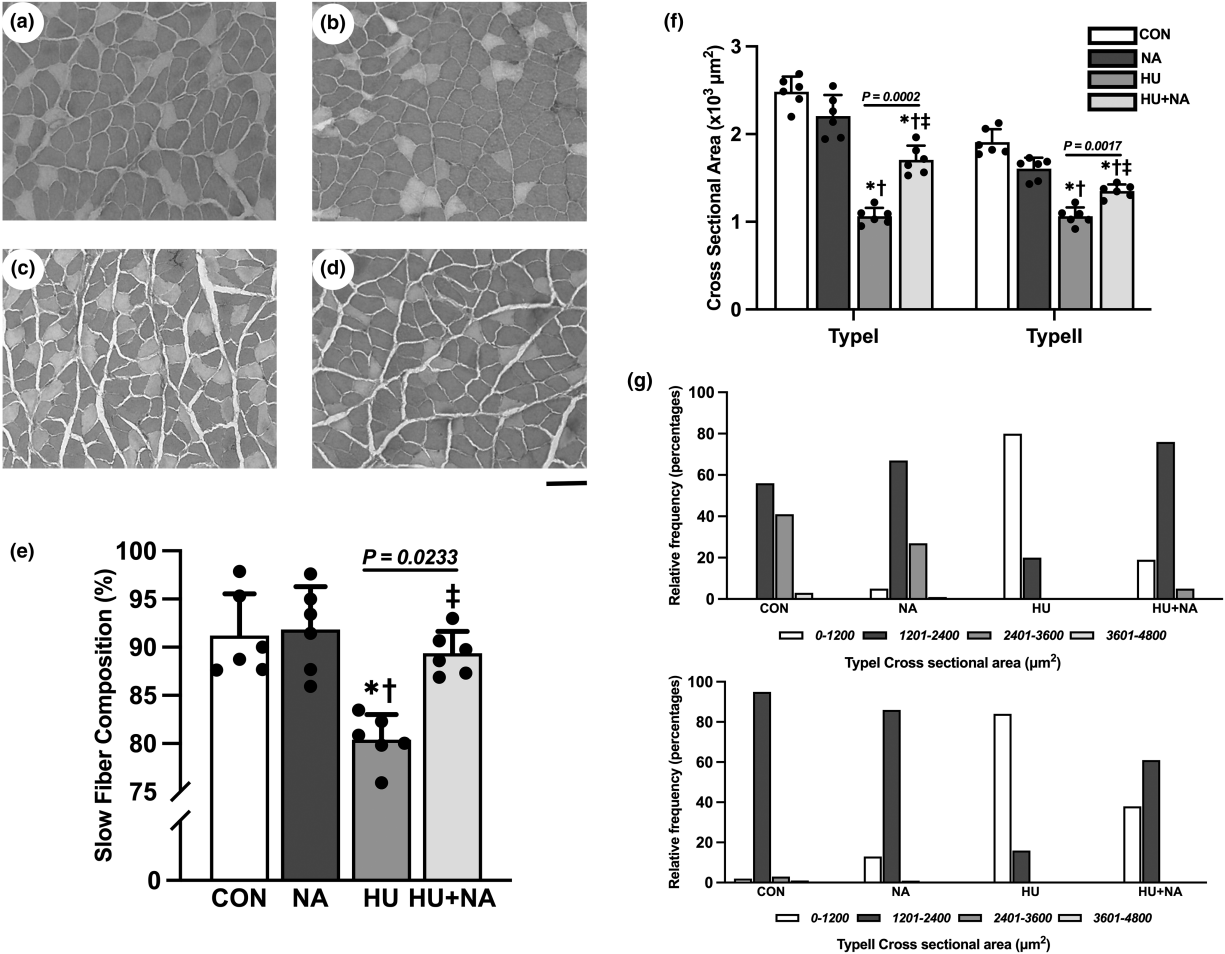
Transverse sections were stained for adenosine triphosphate (ATPase) (a–d, PH:4.2) and slow fiber composition (e) of the soleus muscles. The cross‐sectional area (f) and the distribution of myofiber size in soleus muscles (g). CON (a), NA (b), HU (c), HU + NA (d). Bar = 100 μm. Values are means ± SD (*n* = 6). CON, control; NA, Niacin; HU, hindlimb unloaded; HU + NA, hindlimb unloaded plus Niacin; Light: type II; Dark: type I. The symbols *, † and ‡ indicate significant differences from the CON, NA and HU groups, respectively, at *p* < 0.05. NA treatment attenuated the HU‐induced process of a slow to fast muscle‐type transformation.

It was found that the number of type I fibers in the HU group was significantly lower than that in the control group, while the number of type I fibers in the HU + NA group was significantly higher than that in the HU group. Thus, type II fibers were completely different from type I fibers in terms of composition (Figure 1e).

The average cross‐sectional area of both type I and type II muscle was significantly increased after NA supplementation compared with the HU group. (Figure 1f) The distribution of fiber sizes in HU group was observed to be shifted toward smaller fibers (0–1200 μm^2^). NA supplementation in HU + NA group prevented this shift, with fiber sizes ranging from 1201 to 2400 μm^2^. (Figure 1g).

### 3.3 Capillary‐to‐fiber ratio

**FIGURE 2:**
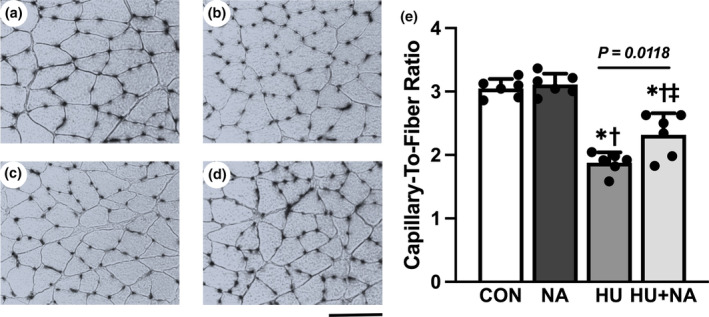
Transverse sections stained for alkaline phosphatase (a–d) and C/F ratio (e) of the soleus muscles. CON (a), NA (b), HU (c), HU + NA (d). Capillaries were visualized as black dots. Bar = 100 μm. Values are means ± SD (*n* = 6). CON, control; NA, Niacin; HU, hindlimb unloaded; HU + NA, hindlimb unloaded plus Niacin. The symbols *, † and ‡ indicate significant differences from the CON, NA and HU groups, respectively, at *p* < 0.05. HU harmed the capillary network while NA treatment prevented the capillary regression in the skeletal muscle.

Despite the fact that C/F ratios were significantly lower in the HU groups than the control group, the value in the HU + NA group was significantly higher than that in the HU group (Figure 2). In both the control and NA groups, the C/F was not significantly different.

### 3.4 Capillary diameter and volume

**FIGURE 3:**
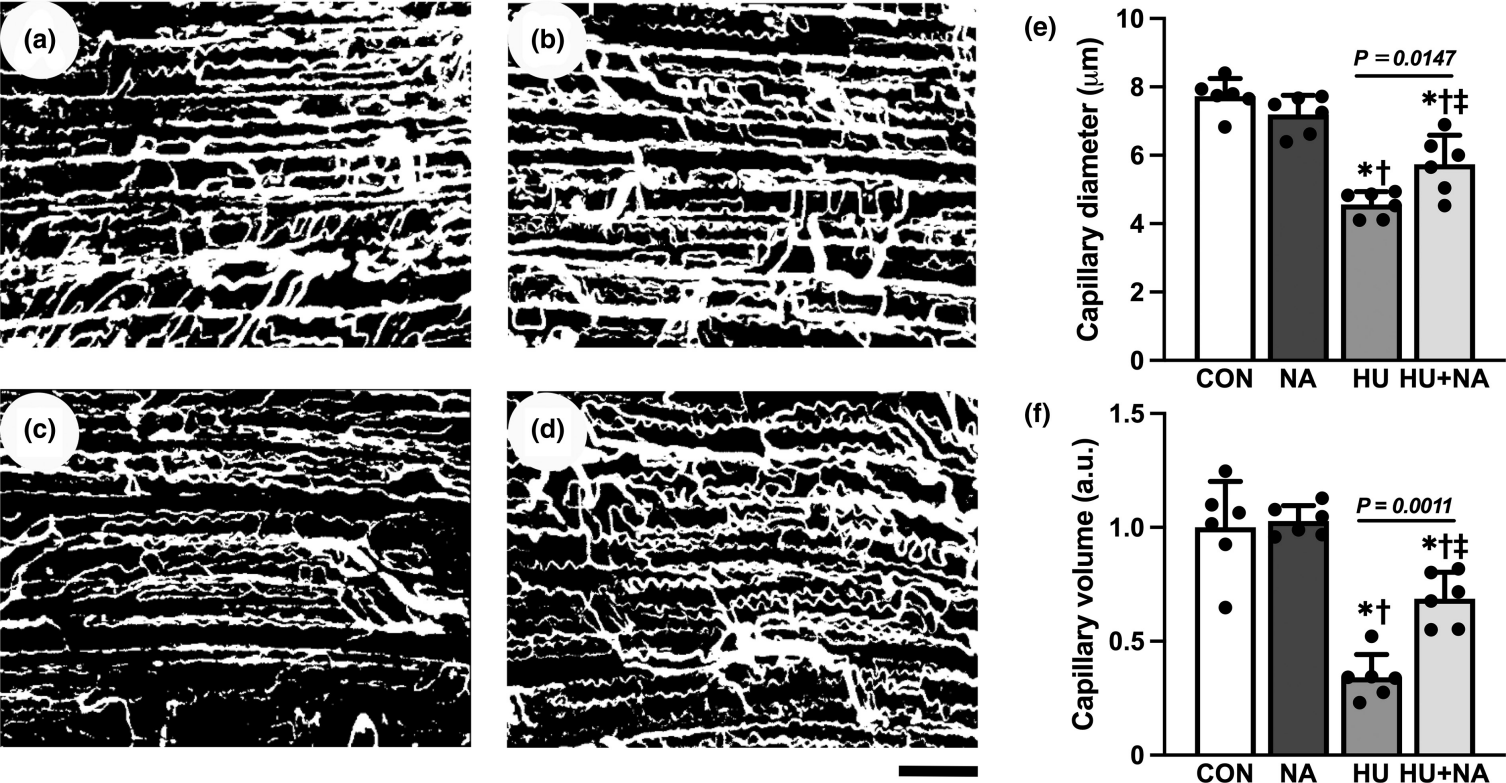
The three‐dimensional (3D) capillary architecture of the soleus muscle of a rat in the CON (a), CON+NA (b), HU (c) and HU + NA (d)as shown by confocal laser scanning microscopy. Scale bar: 100 μm. Figures (e) and (f) depict the mean capillary luminal diameter and capillary volume, respectively, in the soleus muscle of each group. Values are means ± SD (*n* = 6). The symbols *, † and ‡ indicate significant differences from the CON, NA, and HU groups, respectively, at *p* < 0.05.

**FIGURE 4:**
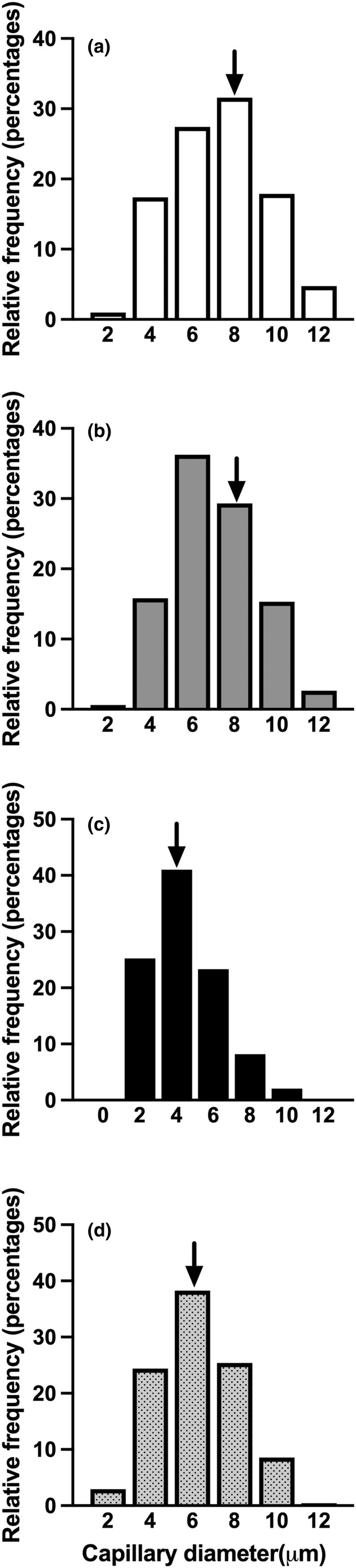
Frequency distribution of capillary luminal diameter in the CON (a), CON+NA (b), HU (c), and HU + NA (d) groups, denoted by arrows indicating the median diameter.

The representative confocal laser scanning microscopic images of 3D capillary architecture in each group are shown in Figure 3 (a–d). The frequency distribution of capillary luminal diameter in the HU and HU + NA groups indicated a shift toward capillaries with reduced diameter compared to the CON and NA groups. Notably, the histogram of the HU + NA group appeared to have a higher distribution than that of the HU group (Figure 4a–d) The mean capillary diameter and volume in the HU group were significantly lower than those in the CON, NA, and HU + NA groups. (Figure 3e,f).

### 3.5 Mitochondrial enzyme activities

**FIGURE 5:**
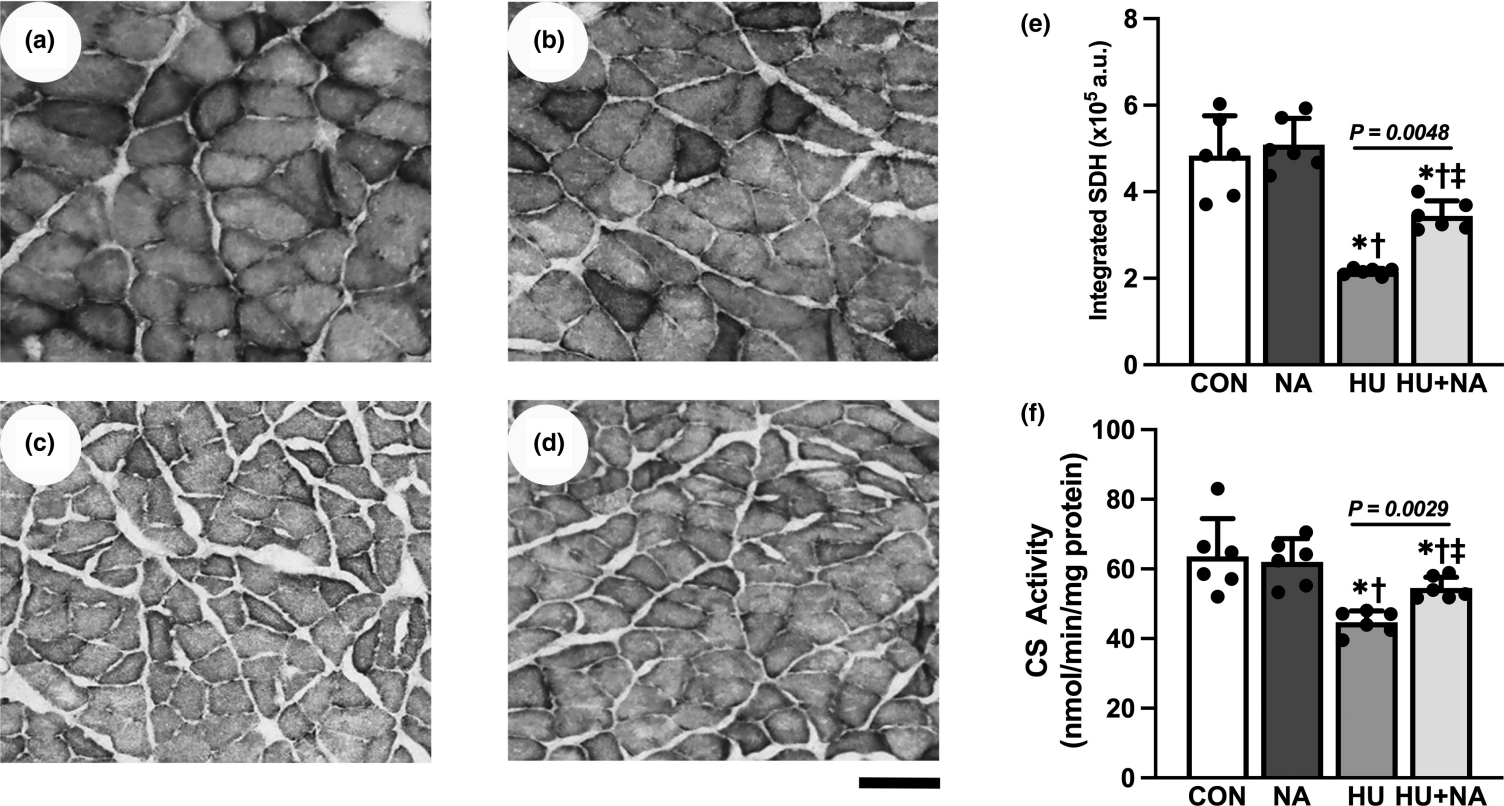
Transverse sections stained for succinate dehydrogenase (SDH) (a–d), Integrated SDH activity (e) and CS activity (f) of the soleus muscles. CON (a), NA (b), HU (c), HU + NA (d). Bar = 100 μm. Values are means ± SD (*n* = 6). CON, control; NA, Niacin; HU, hindlimb unloaded; HU + NA, hindlimb unloaded plus Niacin. The symbols *, † and ‡ indicate significant differences from the CON, NA and HU groups, respectively, at *p* < 0.05. The activity of mitochondrial oxidase was reduced in HU and improved by NA treatment.

Representative images of SDH staining for each group are presented in Figure 5 (a–d). The integrated SDH activity and CS activity, which reflect the total mitochondrial enzymes, was significantly lower in the HU and HU + NA groups compared to the CON and NA groups. Additionally, the value in the HU + NA group was significantly higher than that in the HU group, which indicates that mitochondrial oxidative activity was reduced in the HU group and improved with NA treatment.

### 3.6 PGC‐1α protein expression level

**FIGURE 6:**
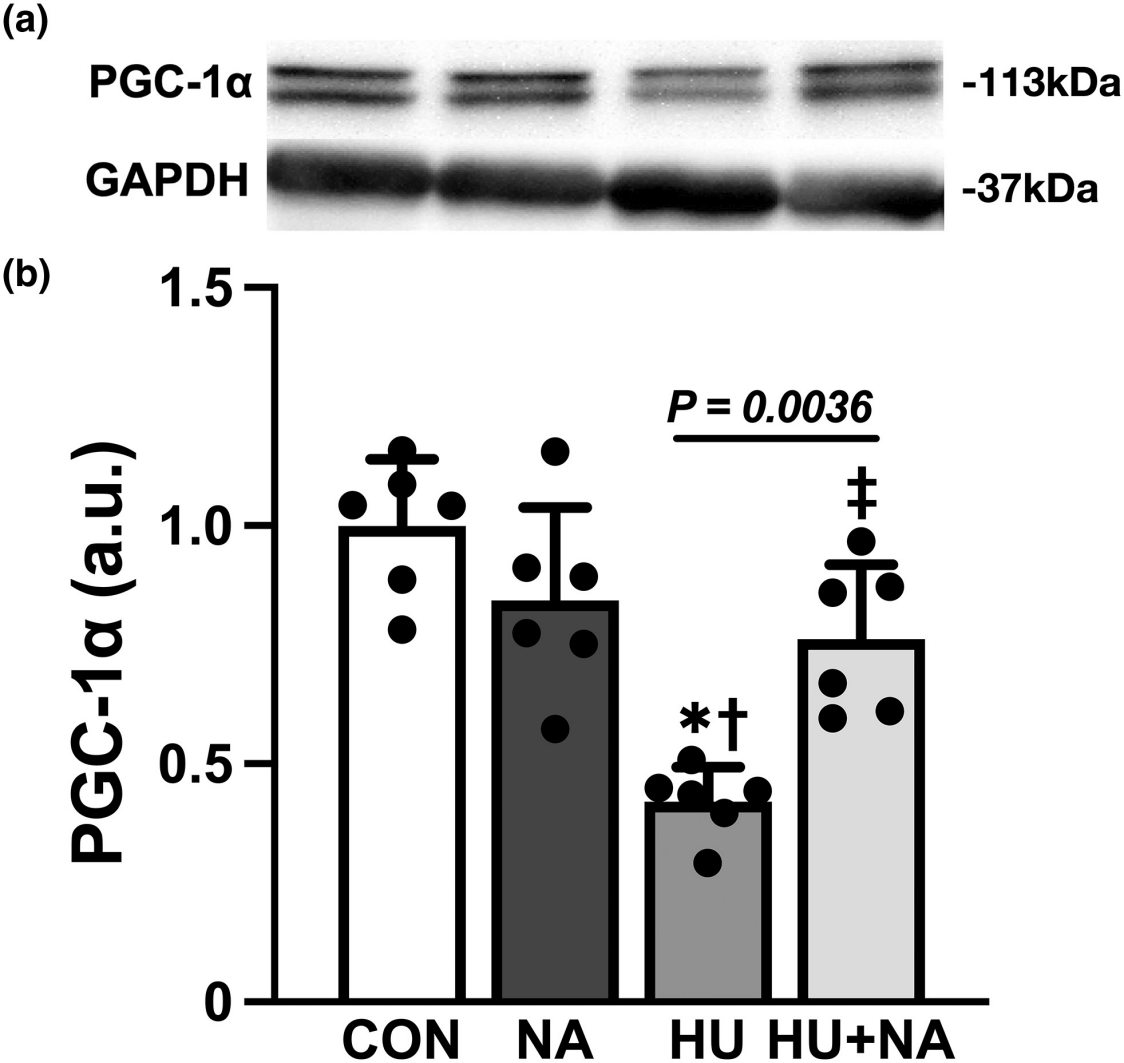
Effect of Niacin (NA) on PGC‐1α protein expression levels in soleus muscle. Representative western blots (a) and PGC‐1α protein expression levels (b) in the soleus muscle. Values were calculated as the fold changes relative to the control group. Values are expressed as mean ± SD. The symbols *, † and ‡ indicate significant differences from the CON, NA and HU groups, respectively, at *p* < 0.05. NA treatment attenuated the HU‐induced decrease of the expression of PGC‐1α protein.

Although the expression of PGC‐1α protein in the HU group was significantly lower than that in the control group, the level in the HU + NA group was significantly higher than that of the HU group (Figure 6). Additionally, there was no difference between the levels of the control, NA, and HU + NA groups.

## 4 DISCUSSION

The key discovery of this study is that NA supplementation effectively prevented a decline in capillary luminal diameter and capillary volume, as well as capillary number by preserving the expression of metabolic factor within the unloaded rat soleus. Moreover, NA supplementation counteracted the muscle fiber transition from type I, also known as oxidative, to type II, also known as glycolytic, induced by unloading. These findings suggest that NA helps maintain mitochondrial metabolic activity in muscle fibers and prevents capillary regression during muscle atrophy caused by unloading.

According to previous study, 2 weeks HU induced capillary regression, manifesting as both a decrease in capillary number and a reduction in capillary luminal diameter and volume in the soleus muscle (Kanazashi et al., 2020). Additionally, HU is also linked to a significant reduction in muscle mass and fiber cross‐sectional area, a type I muscle fiber to type II muscle fiber phenotype transformation, CS activity and a decrease in integrated SDH in the soleus muscle fiber. SDH is part of both the citric acid cycle and the respiratory electron transfer chain, functioning as an oxidative enzyme in the TCA cycle of the mitochondrial matrix that synthesizes ATP (Rutter et al., 2010). CS activity serves as an established biomarker for mitochondrial density In skeletal muscle (Vigelsø et al., 2014). Consequently, integrated SDH and CS activity can be used as an indicator of mitochondrial oxidative potential. Kanazashi et al. (2019) and Wesolowski et al. (2023) demonstrated that integrated SDH and CS activity in skeletal muscle decreased when muscle activity was inhibited by HU. This implies that reduced integrated SDH activity and CS activity in muscle fibers signifies a decrease in mitochondrial number and function in unloaded skeletal muscles. Taken together, these findings align with the observations made in the current study.

NA is known to be an effective NAD+ booster in mammals. Previous research has demonstrated that NA promotes muscle mitochondrial biogenesis and enhances muscle strength in humans with myopathy (Pirinen et al., 2020). Thus, NA supplementation's NAD+ boosting function can protect against mitochondrial dysfunction caused by unloading.

In the current study, our primary focus was directed toward the analysis of morphological changes in the capillarization of rat skeletal muscle. The capillary diameter and volume, as well as the capillary number in the HU + NA group significantly increased compared with those in the HU group. Capillary volume plays a vital role in expanding the capillary bed, facilitating metabolic exchange by increasing capillary vessel numbers (Fujino et al., 2012). Hence, the increased capillary diameter and volume resulting from NA supplementation may enhance oxygen delivery capacity, along with expanding the microvascular bed for efficient exchange. In addition, NA intake improved the decrease in integrated SDH and CS activity in soleus muscles caused by unloading. Furthermore, NA counteracted the muscle fiber transition from oxidative to glycolytic fibers induced by unloading. Because angio‐adaptation is closely related to the intensity of the oxidative metabolic profile and the composition of muscle fiber types (Hudlicka, 1992; Kanazashi et al., 2014), our possible explanation for these results is that increased mitochondrial metabolic activity enhanced oxygen supply to the soleus muscles and caused angiogenesis in the skeletal muscles (Charifi et al., 2004). Based on the above, we concluded that NA supplementation appears to alleviate capillary regression in the soleus muscle due to muscle atrophy by activating mitochondrial metabolism.

In addition, NA during unloading resulted in preventing to decrease PGC‐1α protein expression levels. PGC‐1α assumes a significant role as a critical regulator of muscle oxidative capacity (Geng et al., 2010). It has been suggested that the expression level of PGC‐1α decreases under conditions of muscle inactivity, such as HU (Nagatomo et al., 2011), while it conversely increases in response to muscle activation, such as exercise (Olesen et al., 2010). Our prior investigations have provided evidence of a decline in PGC‐1α expression and diminished SDH activity within the rat soleus muscle as a consequence of HU (Kanazawa et al., 2014). Furthermore, numerous in vivo studies have reported the upregulation of PGC‐1α following HU. (Cannavino et al., 2015; Liu et al., 2014), consequently leading to mitochondrial biogenesis within the muscles. These results suggest that PGC‐1α plays a crucial role in the muscle mitochondria adaptation response to unloading, which is consistent with the findings of this study. In addition to its role in activating mitochondrial metabolism, PGC‐1α is widely recognized as a potent inducer of angiogenesis in skeletal muscle. Mice lacking PGC‐1α in skeletal muscle failed to increase capillary density in response to exercise (Chinsomboon et al., 2009), indicating the powerful ability of PGC‐1α to activate angiogenesis in skeletal muscle. These findings underscore the critical role of PGC‐1α in mediating angiogenic adaptations during skeletal muscle unloading, suggesting that NA supplementation contributes to 3D capillary remodeling within the soleus through the upregulation of PGC‐1α.

In this study, we demonstrated that NA supplementation prevents the decrease in mitochondrial metabolic activity and capillary regression in soleus muscles following HU. Our results suggest that NA supplementation can help mitigate the loss of muscle endurance resulting from HU. In terms of NA intake, a single dosage exceeding 7000 mg/kg in rats is considered acutely toxic (LD50). However, the 750 mg/kg dosage administered in this experiment is significantly lower than the toxic threshold and is deemed safe for rats. For humans, an intake exceeding 3000 mg/day may lead to adverse effects on the body. (Mosher, 1970) Previous studies involving human subjects demonstrated that NA doses ranging from 700 to 1000 mg/day effectively enhance muscle performance in cases of Adult‐Onset Mitochondrial Myopathy. (Pirinen et al., 2020) Therefore, a moderate intake within this range, specifically 700–1000 mg/day, may be considered appropriate for adults.

This study has some limitations. First, our investigation focused exclusively on female rats. The primary objective was to examine the influence of NA supplementation on capillary regression, emphasizing its effects on mitochondrial biogenesis and the expression of PGC‐1α. It is acknowledged that both testosterone and estrogen can influence the mitochondrial pathway through PGC‐1α (Yin et al., 2021). Additionally, Mortreux et al. (2021) reported that following 14 days of hindlimb suspension, female rats exhibited more stable hormonal levels compared to their male counterparts. Given the observed gender‐specific responses to disuse, future studies should include investigations into male rats. Second, the effect of NA on muscle function, such as contractile function, in HU rats has not been confirmed. Furthermore, while our study on limb immobilization using the head‐down tilt model has provided valuable insights into skeletal muscle physiology, it is crucial to recognize limitations, especially considering potential cardiovascular effects observed by Menon and Thomason (1995). Further investigation is needed to address these limitations and determine if similar cardiovascular effects are associated with the 2 weeks of HU in our study. Finally, the mechanism underlying NA‐induced protection against PGC‐1α signaling activation were not fully investigated. Hence, future work should try to focus on elucidating the molecular mechanisms underlying NA‐induced protection against PGC‐1α signaling.

In conclusion, we discovered that NA supplementation is an effective treatment for preventing capillary regression in muscles with atrophy and may be proven to be beneficial for patients who cannot engage in normal activities to maintain the capillary network and mitochondrial metabolism of muscle fibers during physical inactivity. It is important to note that medicinal NA may cause a harmless side effect called NA flush, which typically subsides after an hour. Our findings suggest that NA holds potential as a therapeutic strategy for treating skeletal muscle vasculopathy resulting from disuse‐induced muscle atrophy.

## AUTHOR CONTRIBUTIONS

H.L., N.M. and H.F. conceived and designed the experiments. H.L., JH.X., H.P., R.N.and T.H. performed the experiments. H.L., N.M., H.K., R.N. and H.F. analyzed the data. N.M., H.K. and H.F. contributed by providing reagents, materials and analysis tools. H.L., N.M., H.K. and H.F. interpreted the data and wrote the paper. All authors approved the final version of the manuscript.

## CONFLICT OF INTEREST STATEMENT

It is declared that the authors have no conflicts of interest.

## ETHICS STATEMENT

This study received approval from the Institutional Animal Care and Use Committee and was conducted in accordance with the Kobe University Animal Experimentation Regulations. All experiments adhered to the National Institutes of Health (NIH) Guidelines for the Care and Use of Laboratory Animals (National Research Council 1996).

## Data Availability

The data that support the findings of this study are available from the corresponding author, [H.F.], upon reasonable request.
